# Breast and prostate cancer survivors’ understanding of risk and management of cardiovascular and musculoskeletal side effects of treatment: findings from focus groups

**DOI:** 10.1007/s00520-025-09642-z

**Published:** 2025-06-21

**Authors:** Jack Dalla Via, Chris R. Andrew, Brenton J. Baguley, Nina Stewart, Jonathan M. Hodgson, Joshua R. Lewis, Mandy Stanley, Mary A. Kennedy

**Affiliations:** 1 School of Medical and Health Sciences, Nutrition and Health Innovation Research Institute, Edith Cowan University, Perth, WA Australia; 2 Institute for Physical Activity and Nutrition, School of Exercise and Nutrition Sciences, Deakin University, Geelong, VIC Australia; 3 Allied Health Research, Peter MacCallum Cancer Centre, Melbourne, VIC 3051 Australia; 4 GenesisCare, Perth, WA Australia; 5 Medical School, The University of Western Australia, Perth, WA Australia; 6 School of Medical and Health Sciences, Edith Cowan University, Perth, WA Australia

**Keywords:** Cancer, Survivorship, Treatment side effects, Cardiovascular, Musculoskeletal

## Abstract

**Purpose:**

Survival after a cancer diagnosis is improving, increasing the importance of understanding and managing long-term treatment-related adverse effects. This study aimed to understand breast and prostate cancer survivors’ understanding of how cancer treatment may affect cardiovascular and musculoskeletal health.

**Methods:**

Australian breast and prostate cancer survivors treated with therapies with known cardiovascular adverse effects were recruited via a private cancer care provider. Participants completed an online background questionnaire, then participated in a focus group. Focus groups were recorded, transcribed, then analysed using reflexive thematic analysis.

**Results:**

In total, 26 cancer survivors (15 breast, 11 prostate; mean age 67 years) participated in one of seven focus groups. Three major themes were developed: 1) *Focus is on the here and now, not the future*—participants were infrequently told that their cancer treatment can have negative long-term effects; 2) *Wanting individualised delivery of side-effect information*—participants received varying types and amounts of information about side effects, but it was not delivered in a way that best suited them; and 3) *Left wondering how to best manage side effects*—few participants were provided with information about how to manage long term side effects, despite wanting this information.

**Conclusion:**

Important information about long-term side effects of cancer treatment, and how to manage them, was inconsistently provided to breast and prostate cancer survivors. Information about long-term treatment side effects should be delivered in a flexible, individualised way to better enable cancer survivors to understand the risk and engage in preventative health behaviours.

**Supplementary Information:**

The online version contains supplementary material available at 10.1007/s00520-025-09642-z.

## Introduction

Cancer incidence is increasing with a growing and ageing population, however survival rates are also improving meaning more cancer survivors are living longer following a diagnosis [1, 2]. This is particularly evident in breast and prostate cancer, among the most common types, in which more than 90% of those diagnosed live at least 5 years post diagnosis [1]. Common therapies for breast and prostate cancer, including radiotherapy, chemotherapy and/or hormone therapy, have long-term treatment-related adverse effects that include, but are not limited to, accelerated muscle and bone loss, impaired body composition, and poorer cardiovascular health [3–6]. Consequentially, cancer survivors are shown to have an increased risk of important clinical endpoints relating to musculoskeletal health and cardiovascular disease (CVD). Among cancer survivors alive 5 years after diagnosis, CVD-related deaths are shown to exceed cancer-related deaths from 13 years after cancer diagnosis [7]. Similarly, breast and prostate cancer survivors are shown to have a 15–80% increased risk of falls compared to cancer-free controls, and a 21–59% increased risk of fracture, which remains increased beyond five years post diagnosis [8–10]. Raising awareness of the long-term consequences of cancer treatment is important to help attenuate musculoskeletal and cardiovascular decline and reduce these risks.

An Australian position statement on cancer survivorship care emphasises the need for individualised care pathways that continue beyond cancer diagnosis and treatment [11]. Furthermore, consensus guidelines for patient-clinician communication in oncology recommend that clinicians provide individualised information, and check patient’s understanding of potential benefits and burdens of treatment [12]. Cancer survivors need to understand long-term treatment-related adverse effects and how they can be mitigated into survivorship. However, whether these issues are consistently discussed with cancer survivors during and following treatment, and when they are most receptive to this information, remains unclear. In a recent systematic review, one third of cancer survivors aged over 60 years believed that while information received about the cancer itself and treatment options was satisfactory, information about treatment side-effects was insufficient [13]. This was despite side effect information being highly important to them [13]. Specifically regarding CVD risk, qualitative interviews revealed that Australian cancer survivors have limited awareness of elevated CVD risk following cancer [14].

Understanding long-term side effects of cancer treatment may contribute to cancer survivors adhering to evidence-based recommendations, such as exercise and dietary intervention [15], to mitigate these effects throughout survivorship. In women diagnosed with breast cancer, education about short-term side effects of chemotherapy and how to manage them is shown to improve symptoms and self-care behaviours [16]. Similarly, increased knowledge of chemotherapy side effects is shown to be associated with better self-care practices, including diet and exercise behaviours, to manage the side effects among Indian cancer survivors [17]. Therefore, it is likely that understanding what information cancer survivors receive about long-term treatment-related side effects, and opportunities to improve the delivery of this information, will help to develop more effective supportive care for people with cancer.

There is a clear need to understand what cancer survivors are told about possible long-term treatment side effects, and about how they can be managed. This will help to improve long-term survivorship care and reduce the burden of important adverse effects that may not manifest until years following treatment. This qualitative study aims to explore the information provided to breast and prostate cancer survivors about cardiovascular and musculoskeletal side effects of cancer treatment. This study was the second aim of a previously published focus group study [18] that explored exercise and diet support received by breast and prostate cancer survivors during and following treatment.

## Methods

### Study design

Results relating to the first aim of this qualitative descriptive study have been reported previously, including a detailed description of the methods used [18]. Therefore, below is an overview of the relevant methods. This study used focus groups to allow for open discussion and interaction between participants, and to help uncover insights that would not have been revealed otherwise [19]. The researchers took a relativist ontology and a subjectivist epistemology, valuing the perspective of the participants and ensuring their perspective is foregrounded in the interpretation [20]. Ethical approval was granted by the Edith Cowan University Human Research Ethics Committee (Project number 2021–02863-DALLAVIA). This study adheres to the consolidated criteria for reporting qualitative research (COREQ) [21].

### Study sample

#### Eligibility

Breast and prostate cancer survivors aged 18 years or older who had completed active treatment (ongoing hormone therapy acceptable) with therapies known to have cardiovascular and/or musculoskeletal adverse effects were eligible to participate. This included radiotherapy, chemotherapy and/or aromatase inhibitor therapy for breast cancer, and androgen deprivation therapy and/or chemotherapy for prostate cancer.

#### Recruitment

Participants were recruited via a large private cancer care provider in Perth, Western Australia using purposive sampling. An invitation was sent to 354 potential participants (183 breast cancer survivors, 171 prostate cancer survivors) who had completed active treatment from July 2020 to June 2021. All interested participants who contacted the lead investigator were screened for eligibility via telephone, and eligible participants provided informed consent prior to completing the online background questionnaire or attending a focus group.

### Data collection

#### Questionnaire

Background demographic, medical, physical activity and diet information about participants was collected using an online questionnaire (Qualtrics, Utah, USA) completed prior to the focus group. Medical questions collected both general medical information, as well as cancer-specific information (diagnosis and treatment information).

#### Focus groups

Breast and prostate cancer survivors participated in separate in-person focus groups, held at the Royal Perth Hospital Research Foundation building in Perth in December 2021, moderated by JDV (male, PhD, postdoctoral research fellow, experience in conducting research with cancer survivors). One observer joined each focus group to record field notes [22]. Only the moderator, observer and participants attended each focus group. A focus group semi-structured question guide developed by the research team was used to guide each conversation (provided as supplementary information). The focus group guide was not pilot tested for practical reasons, but small iterative changes were made following the first focus group to improve the clarity of the questions and relevance of the discussion. Audio of each focus group was digitally recorded, then professionally transcribed (Digital Transcripts, Victoria, Australia). Participants received a $30 (AUD) gift voucher as reimbursement and were compensated for any parking expenses associated with attending the focus group.

### Data analysis

Responses to demographic and medical questions in the online questionnaire were summarised descriptively. Focus group transcripts were imported into NVivo (version 12, QSR International) for analysis, using reflexive thematic analysis, following the six steps of reflexive thematic analysis [23, 24]. Participants were not asked to review transcripts as they were transcribed verbatim. Two investigators (JDV, CA) initially coded the same single focus group, compared and discussed the results with a third investigator (MK) to ensure consistency, then coded the remaining focus group transcripts. Codes were collated into categories, then themes were developed in consultation with other investigators (MK, MS). The themes were further refined by ongoing discussion and reflection among the authors to ensure they answered the research question and accurately represented the data. Participants did not provide feedback on the results of the thematic analysis.

## Results

### Study sample

In total, 26 cancer survivors (15 breast cancer, 11 prostate cancer; mean ± SD age 66.7 ± 11.7 years; mean ± SD time since diagnosis 16 ± 8 months) participated in a focus group. Of the 354 cancer survivors initially contacted, 36 (10%) expressed interest and were screened for eligibility. Of these, 28 were eligible, interested in participating, and provided informed consent. Two of these participants did not attend the focus group (one for unknown reasons, one due to unforeseen family circumstances). Seven focus groups (four breast cancer, three prostate cancer) with three or four participants each lasting 54 to 107 min were completed. Participant characteristics are reported in detail previously [18]. Briefly, 40% of breast cancer survivors had stage I cancer, while most prostate cancer survivors (82%) did not know the stage of their cancer. All breast cancer survivors had been treated with surgery and radiotherapy, and most (87%) were treated with hormone therapy. All prostate cancer survivors had completed radiotherapy as well as previous or ongoing androgen deprivation therapy. A family history of heart disease was the prevalent health concern reported most often (47%) among breast cancer survivors. Most prostate cancer survivors reported having hypertension (73%) and hypercholesterolemia (64%).

### Themes

Three themes were developed from the focus group discussions and are described below. Both participant groups shared similar discussion points and experiences, so themes apply to both breast and prostate cancer survivors unless specified otherwise. Based on these discussions with cancer survivors, a series of considerations regarding the content and delivery of side effect information are presented in Table 1. Consideration of these factors, where possible and practical, may help to more effectively meet the needs of cancer survivors when providing cancer survivors with information about treatment-related side effects.

**Table 1:** Consumer-informed considerations for improving the content and delivery of information about cancer treatment-related side effects

Content	
**Short- and long-term**	Information about long-term side effects cancer survivors may experience should be provided. This should be in addition to the information they more often receive about short-term side effects of cancer treatment. If being sent for testing related to long term side effects (e.g., bone density scans because of anticipated bone loss), the reason for this testing should be explained to cancer survivors.
**Individualisation**	Side effect information should be specifically relevant to each cancer survivor and what they can expect, as opposed to generic information based on cancer site or treatment type.
**Management**	Support and information about how to manage possible side effects should be provided. This is especially important for behavioural management of physical and psychosocial side effects, which is more often lacking.
**Delivery**	
**Flexibility**	Flexibility in the how side effect information is delivered to cancer survivors, taking into consideration individual preferences and needs, may help them to better understand the information provided. Specifically, there should be flexibility in how, what, when, and by who information is delivered.
* How*	Cancer survivors’ preferences for how they would like side effect information provided to them (e.g., verbal [face-to-face or telehealth], written, video, etc.) should be considered. Cancer survivors should have the opportunity to discuss side effects with their medical team.
* What*	The type and amount of side effect information should be tailored to each cancer survivors’ preferences, so it best suits their individual needs and can be better understood.
* When*	The timing for providing side effect information should be discussed with each cancer survivor, so they can best receive and comprehend it. This timing may differ for each individual. Side effect information should be provided proactively, rather than reactively after a cancer survivor brings up a side effect they are experiencing. Side effect information should be reinforced regularly, not just provided once at or shortly after diagnosis. This is especially relevant for longer-term side effects.
* Who*	No preference of healthcare professional that provides side effect information, provided they have sufficient time to do it meaningfully.

#### Theme 1: Focus is on the here and now, not the future

Participants reported receiving some form of information about possible treatment side effects. However, the most common side effects discussed were those typically experienced in the short-term during treatment for breast cancer (e.g., nausea, fatigue, radiation burns) or for prostate cancer (e.g., hot flushes, incontinence).

“Tiredness. I was told about the tiredness. Um, but that was the main one” (Participant 7, Focus Group 4, Breast cancer, 63 years of age)

“I think the main ones they told you were the hot flushes. That was about it.” (P17, FG5, Prostate cancer, 67 years)

Few participants were told about long-term side effects they may experience in the years following completion of treatment, such as musculoskeletal decline and increased cardiovascular disease risk.

“I don't recall anything long-term” (P2, FG3, Breast cancer, 52 years)

“I never had that, nothing about the heart was checked, or bone density, nothing” (P10, FG2, Breast cancer, 34 years)

“At no time did anyone tell me that it can affect your heart.” (P9, FG1, Breast cancer, 66 years)

“Nothing’s been said to me that it [treatment] could affect my heart.” (P18, FG7, Prostate cancer, 79 years)

The few participants who were told that increased cardiovascular disease risk was a possible consequence of cancer treatment were mostly women with breast cancer undergoing radiotherapy.

“They said that because it is overlapping, when you're doing the radiotherapy, in the long run my heart may have some issues, but this chance is a bit low.” (P4, FG1, Breast cancer, 60 years)

Several participants completed bone density scans as part of their cancer care, but few of them were told why this was necessary and how it related to the treatment they received.

“I had to undergo bone density tests before but… I have never been told anything about bone density.” (P20, FG6, Prostate cancer, 78 years)

#### Theme 2: Wanting individualised delivery of side-effect information

Information provided about possible treatment-related side effects varied among cancer survivors, in terms of the volume and type of information received as well as how it was delivered. For example, some participants received a small amount of vague information, while a large list of possible side effects of a given treatment type was communicated to others.

“I thought they were very vague on possible side effects. They didn’t tell me a great deal at all.” (P17, FG5, Prostate cancer, 67 years)

“I wasn’t told anything. I was given a whole lot of booklets to read.” (P20, FG6, Prostate cancer, 78 years)

“They give you a lot of things and when you go for your first chemo, they tell you all the side effects and everything” (P1, FG2, Breast cancer, 76 years)

Participants often reported receiving large amounts of written information regarding possible side effects and what may be expected during treatment, and their perceptions of this varied. Some were overwhelmed by the volume of written information they received, some were happy, while others did not read it thoroughly.

“There is so much information that we’re given as well that sometimes it was a bit overwhelming and it’s like, oh my God. You’ve got this website, that website. You’ve got this app, you’ve got that app. You’ve got this person to call, that person. This agency, that agency, and it is very overwhelming” (P11, FG2, Breast cancer, 46 years)

“I was pretty happy with what they gave us. I thought it was enough for me.” (P23, FG5, Prostate cancer, 81 years)

“They also gave me a book about this thick and a whole pile of pamphlets and things which I kind of flicked through, but I really didn't read them.” (P6, FG3, Breast cancer, 75 years)

Written information alone was not considered sufficient by most participants. They consistently reported wanting the option to discuss side effects with their medical team to help support the written information provided and to answer any questions they may have.

“I think that you – you need to have someone you can approach to get information. You can read so much but…” (P18, FG7, Prostate cancer, 79 years)

Consistently though, written and verbal information that was provided was general in nature. Many participants perceived this lack of specificity or definitive information as the medical team being uncertain.

“It was more the booklets really and just ‘possibles’. You know, ‘this is possible, but it might not affect you’. ” (P14, FG4, Breast cancer, 60 years)

“ ‘That’s normal’. ‘How long’s this going to last?’, ‘Oh, it depends’… I find you don’t really get a straight answer.” (P19, FG5, Prostate cancer, 72 years)

Some participants perceived the provision of side-effect information to be reactive. Participants reported being asked if they were experiencing any treatment side-effects, and if they answered that they were not, then no further information about possible side effects or their management was provided.

“[They] would say, “Anything wrong?” And if you said, “No”, that was it.” (P21, FG5, Prostate cancer, 69 years)

               “I had to go out hunting for help.” (P12, FG4, Breast cancer, 61 years)

Nurses were generally considered particularly helpful and well-placed for providing information and answering questions about possible side effects. This was largely due to cancer survivors feeling that they had more time to spend with them, for example when administering hormone injections for prostate cancer or during radiotherapy for breast cancer.

“The oncology nurse was brilliant, she told me what to expect.” (P5, FG1, Breast cancer, 50 years)

“A nurse would come to the house and give me the shot and she explained a lot of the side effects to me.” (P23, FG5, Prostate cancer, 81 years)

“I think the nurses will give you more time too, don’t rush you out. You know, you can ask your questions and they answer your questions.” (P19, FG5, Prostate cancer, 72 years)

#### Theme 3: Left wondering how to best manage side effects

Even though most participants were told about many short-term and occasionally some long-term treatment side effects they may expect, few were provided with information or strategies to combat or manage those side effects. This is despite them wanting this information.

“No management strategy for the side effects” (P2, FG3, Breast cancer, 52 years)

“I wanted some guidance especially from the medical science to give me the opportunity to address [treatment-related side effects] better and improve my health.” (P10, FG2, Breast cancer, 34 years)

If information about managing side effects was provided, it was typically related to more short-term side effects. Management of long-term side effects was rarely discussed, despite participants wanting information on how to prevent long-term side effects.

“I just sort of remember being told if you are tired, just rest.” (P7, FG4, Breast cancer, 63 years)

“They did say there was medicine they could give you if it [hot flushes] was too severe” (P23, FG5, Prostate cancer, 81 years)

“When you’re preparing you for possible side effects of osteoporosis, I think, that could be a bit more of a focus on the bone strength and the preventative sort of stuff.” (P14, FG4, Breast cancer, 60 years)

Some participants took it within themselves to source more information regarding side-effect management. This involved both individual research and support networks investigating what may be beneficial.

“I was sitting on my, you know, iPad for - for hours looking for information.” (P13, FG3, Breast cancer, 63 years)

“I think the family were doing more research than I was. I was a bit too frightened to find out too much.” (P12, FG4, Breast cancer, 61 years)

“I did do a little bit of research into it [exercise]” (P26, FG6, Prostate cancer, 68)

Participants were rarely told how exercise and diet may help them to manage treatment-related side effects, as described previously [18]. This was also true specifically for long-term treatment-related side effects such as impaired cardiovascular and musculoskeletal health. Very few participants received any information about the role exercise and diet can play in mitigating these effects.

## Discussion

This study presents findings related to understanding of long-term side effects of cancer treatment from focus group discussions with Australian breast and prostate cancer survivors. The results show that long term side effects, such as impaired cardiovascular and musculoskeletal health, were discussed less often than short-term side effects. Side effect information overall, including long-term side effect information when provided, was not often delivered in a way that would best suit cancer survivors. Importantly, cancer survivors were infrequently told about management strategies to combat long-term side effects of their cancer treatment. This left many cancer survivors unaware of evidence-based approaches that could improve their health into survivorship.

Participants were often provided with minimal information about long-term side effects of cancer treatment, despite being interested in this. This is also despite cancer survivorship care recommendations emphasising the need for ongoing care in those susceptible to late effects of cancer treatment [11]. A recent systematic review on information needs in older cancer survivors reported that treatment side-effect information provision was considered insufficient despite being highly important to patients [13]. However, perceptions of long-term adverse effects specifically were not reported [13]. Our findings regarding cardiovascular side effects are consistent with those of three Australian qualitative studies with cancer survivors, including those with confirmed cardiotoxicity or attending a cardio-oncology clinic [14, 25, 26]. Participants often reported not being told about cardiovascular risk prior to their cancer treatment, nor strategies to manage cardiovascular risk [14, 25, 26]. While all included prostate cancer survivors were treated with androgen deprivation therapy, which is associated with impaired cardiovascular health [27], participants rarely mentioned this being discussed with them.

Regarding musculoskeletal health, most breast cancer survivors reported having their bone density tested. This largely aligns with global recommendations that all women having hormone therapy for breast cancer undergo bone density testing at the start of treatment [28]. However, despite guidelines recommending bone density testing when commencing androgen deprivation [29], few men reported having bone density tested as part of their treatment and most were unaware of potential treatment-related skeletal adverse effects. Even for the participants who did have bone density tests, most were not often informed about how this was related to their cancer treatment. Importantly, participants received very little information about how to best manage cardiovascular or musculoskeletal health, despite these adverse effects being likely to persist for many years. This issue is likely compounded by ongoing oncology care typically decreasing over time following the completion of active treatment [11, 30], meaning there is even less opportunity for provision of information and management approaches to combat long-term adverse effects. Together, this evidence suggests that cancer survivors may not realise or fully understand the long-term effects of treatment on their cardiovascular and musculoskeletal health. Therefore, they may not appreciate the need for ongoing management approaches, such as exercise and dietary intervention, to mitigate this risk.

Cancer survivors consistently discussed wanting to receive more information about potential long-term treatment-related side effects. More specifically, participants clearly want information about treatment-related side effects to be relevant to them and delivered in a way that they can comprehend, with evident variety in preferences for the type and timing of information and who provides it. These findings are consistent with another Australian qualitative study, where cancer survivors reported diverse preferences for the delivery of information about CVD risk, and approaches to manage this following cancer treatment [14]. It is also consistent with our previously reported findings about exercise and diet support, where support tailored to individual needs was highly desired [18]. Taken together, flexible approaches of information delivery in more than one form, that can be tailored to individual needs and preferences are urgently needed to ensure each cancer survivor better understands the short- and long-term consequences of their cancer treatment, and importantly, what they can do about it. This individualisation should consider the varying needs of different cancer survivors, including factors such as health literacy, social support, and financial means. However, such strategies will not be impactful unless they can be readily translated into clinical practice. Therefore, it is critical that future research considers implementation issues [31, 32] and uses co-design strategies to develop solutions for delivering information that meet the needs of consumers and are practical for health care professionals to deliver [33]. This approach would help to increase the likelihood of achieving meaningful change in clinical cancer care.

Cancer survivors not consistently being told or not understanding how to combat long-term side effects of treatment may experience serious negative consequences years into survivorship. The risk of adverse outcomes such as CVD and fracture is likely exacerbated by a lack of preventative action. Various guidelines detail the clinical management of cancer treatment-related side effects such as cardiotoxicity [34] and bone loss [35], as well as the role of exercise and nutrition in cancer survivorship [15, 36, 37]. However, it appears this information is not consistently reaching the cancer survivors intended to benefit from it. It is well established that making and sustaining changes to health behaviours is difficult, including among cancer survivors who face wide-ranging unique and complex challenges [30, 38]. This is reflected in only 5–23% of cancer survivors adhering to lifestyle behaviour recommendations [39, 40]. Our findings align with previous qualitative work suggesting cancer survivors want to know how they can help themselves by making sustainable health behaviour changes and why it is necessary, but that this information is not consistently or effectively provided to them [14, 30, 41]. This is despite the availability of guidelines that provide oncology clinicians with recommendations for best practice patient-clinician communication [12]. More effectively delivering information about long-term side effects so that cancer survivors understand their importance, then providing tailored information on how to manage those side effects, may provide cancer survivors with both the ‘why’ and ‘how’ that motivates them to engage in appropriate management strategies. This is evident in our previously reported findings in relation to exercise and diet, where cancer survivors reported being more motivated to change their exercise and dietary behaviours if they were told why it would benefit their cancer treatment or combat side effects [18]. This aligns with the COM-B behaviour system, which suggests an individual is more likely to change a behaviour (e.g. engage in strategies to reduce side effects of cancer treatment) if they have the capacity, opportunity and motivation to do so [42]. Overall, our findings suggest there is an opportunity to leverage improved delivery of side effect information into better engagement with management discussions and interventions. This may be particularly important for long-term side effects that are largely asymptomatic (e.g. bone loss, subclinical CVD) but have serious endpoints such as fractures and cardiovascular events [3, 7, 8].

There are several limitations of the current study to consider, as reported in detail previously [18]. Briefly, the findings may not be generalisable to cancer types other than breast and prostate, to cancer survivors treated outside of private cancer care, or those living in rural or regional areas. The results are also not generalisable to other countries but are likely consistent in countries with similar demographic characteristics and/or health care systems. The cancer survivors who volunteered to participate in the study may also have a greater interest in healthy exercise and dietary behaviours than the broader population of cancer survivors. Finally, while the moderator (JDV) disclosed a background in exercise and nutrition research and aimed to not contribute personal thoughts to the discussions, it is possible that this had some influence. However, participant responses were varied and not only what would be considered socially desirable, so it appears unlikely that the moderator influenced discussions. Other than brief conversations during telephone screening and immediately prior to the focus groups commencing, no relationship between the moderator and participants was established.

In conclusion, information about important long-term side effects of cancer treatment, and how to manage them, is inconsistently provided to breast and prostate cancer survivors. Cancer survivors want this information delivered in an individualised way that enables them to understand and action it. Future research investigating flexible, tailored approaches to deliver information about long-term side effects and their management will help to more effectively engage cancer survivors in preventative health behaviours. Considerations of consumer and stakeholder needs will help to translate such approaches into practice and improve cancer survivorship by reducing the burden of the many persisting treatment-related side effects.

## Supplementary Information

Below is the link to the electronic supplementary material.

Supplementary file1 (DOCX 19 KB)

## Data Availability

The data that support the findings of this study are available from the corresponding author upon reasonable request.
