# Supplementary material for: Breast and prostate cancer survivors’ understanding of risk and management of cardiovascular and musculoskeletal side effects of treatment: findings from focus groups
Source: Support Care Cancer. 2025 Jun 21;33(7):608. doi: 10.1007/s00520-025-09642-z (PMC12182484; doi:10.1007/s00520-025-09642-z)
Supplement: Supplementary file 1 — Supplementary file1 (DOCX 19 KB) [file 520_2025_9642_MOESM1_ESM.docx]

**Question list: Understanding of long-term side effects of cancer treatment**

1. **What were you told about possible side effects of your cancer treatment?**

Possible follow-ups:

- *What side effects were you told you could expect?*
- *Could you tell me more about how this information was delivered to you?*
- *Who was it that discussed this with you?*
- *When was this brought up?*
- *How was this information delivered?*
- *How well do you think you understood the information provided?*
- *How regularly has this been brought up to you?*

If only short term side effects discussed:

- *What were you told about any possible long-term side effects of treatment?*

If cardiovascular/musculoskeletal side effects not mentioned by anyone:

- *We’ve discussed several different side effects, but I haven’t heard anyone mention any cardiovascular or musculoskeletal side effects, so related to your heart and blood vessels, muscles and bones. What were you told about this?*

1. **When do you think you were, or would have been, most receptive to hearing information about side effects of treatment?**

Possible follow-ups:

- *Why would this have been the best time for you to receive information about side effects?*

1. **Who do you think you would most like to hear this information from?**

*E.g. your oncologist, a nurse, a cardiologist, your GP.*

Possible follow-ups:

- *Why do you think that is?*

1. **What sort of information about treatment side effects do you think would have helped you the most?**

Possible follow-ups:

- *What content/topics would be of most value to receive information about?*
- *How could this information be delivered to be most helpful?*
